# A cross-sectional observational study showing sebaceous glands differences between psoriatic alopecia and alopecia areata

**DOI:** 10.1016/j.jdin.2024.02.003

**Published:** 2024-02-15

**Authors:** Betty Nguyen, Curtis T. Thompson, Antonella Tosti

**Affiliations:** a Dr. Phillip Frost Department of Dermatology and Cutaneous Surgery, University of Miami Miller School of Medicine, Miami, Florida; b Division of Clinical Sciences, School of Medicine, University of California, Riverside, Riverside, California; c CTA Pathology, Portland, Oregon; d Departments of Dermatology and Pathology, Oregon Health and Science University, Portland, Oregon

**Keywords:** alopecia areata, digital microscopy, hair, histopathology, psoriasis, psoriatic alopecia, sebaceous gland

*To the Editor:* The dermal histopathologic changes in psoriatic alopecia can mimic subacute alopecia areata (AA). These 2 disparate diseases both have follicular miniaturization, a dramatic catagen/telogen shift, and a superficial lymphocytic infiltrate.^1^ Unfortunately, the subacute phase of AA often has a sparse lymphocytic infiltrate and only rarely has the hallmark peribulbar “swarm-of-bees” lymphocytic infiltrate. Thus, it is sometimes not possible to determine whether a patient has psoriasis alone or psoriasis with associated AA. An overdiagnosis of AA may make a patient’s care confusing.

Previous studies have reported sebaceous gland “atrophy” (or complete absence) in psoriatic alopecia^2^ and a small volume^3^; and a normal-to-decreased number of sebaceous glands in AA.^4^ Despite these observations, no one has compared the 2 diseases side-by-side to determine whether additional histologic features exist which can be useful in distinguishing them from each other.

We compared the size and histologic features of sebaceous glands from biopsies with definitive diagnoses of psoriatic alopecia or AA made by 2 of the authors with expertise in hair loss disorders, 1 a clinician and 1 a dermatopathologist. There were 40 Caucasian from a single clinic in Italy, matched as closely as possible by age and gender (20 psoriatic alopecia [average age 43.8 years; 80% female]; 20 AA [average age 45.3 years; 75% female]). A single 4 mm archival biopsy from the margin of each lesion was analyzed for each patient. The transverse sectioning technique allowed evaluation of all histologic levels of the tissue segment. The percentages of large, pale-staining “mature” and small, blue-staining “immature” sebocytes were estimated from cross-sectional images.

Surface areas of sebaceous glands were measured from the largest gland identified using closed-freeform annotation tools built into the Philips IntelliSite Digital Pathology Solutions system.

There was a large difference in the proportions of mature and immature sebocytes. In psoriatic alopecia 29.8% of sebocytes were mature, and the remaining were immature (Fig 1). In AA, in contrast, 87.4% of sebocytes were mature and the remaining were immature (Fig 2) (*P* < .00001).

**Fig 1:**
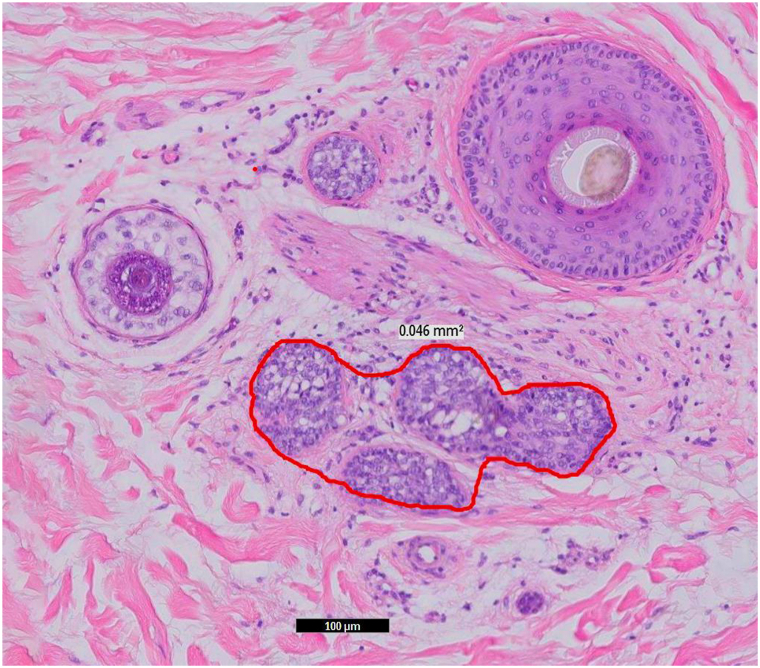
Histopathologic features of a representative sebaceous gland in psoriatic alopecia showing a small sebaceous gland measuring only 0.046 mm^2^ in size and composed of small, “immature” sebocytes. (Hematoxylin-eosin stain; original magnification: ×400.)

**Fig 2:**
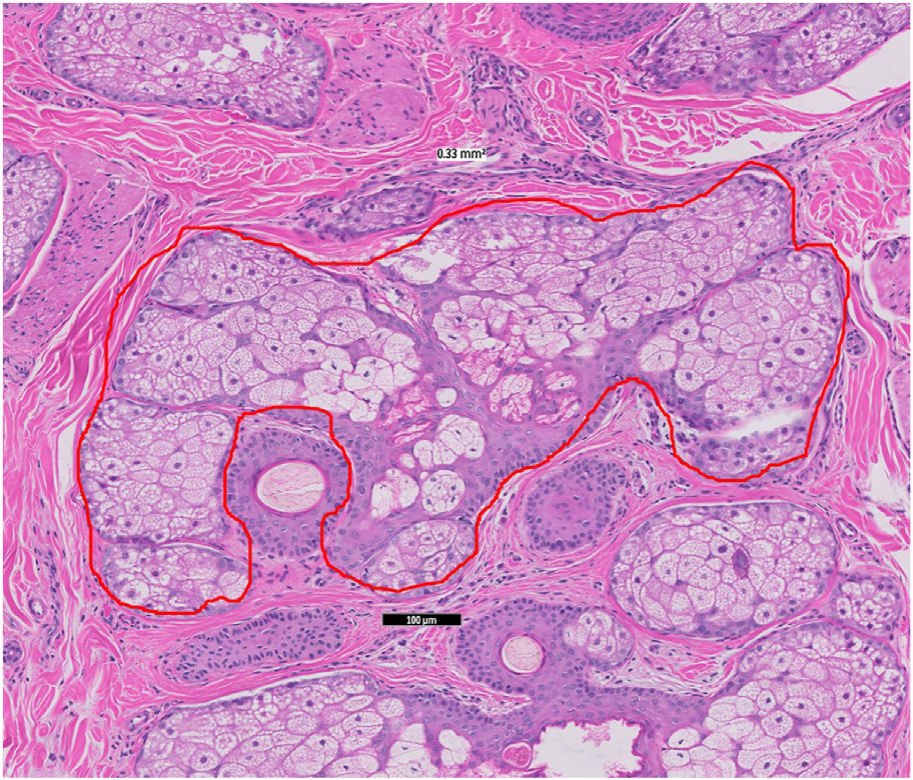
Histopathologic features of a representative sebaceous gland in alopecia areata showing a large sebaceous gland measuring 0.33 mm^2^ in size and composed of large, “mature” sebocytes. (Hematoxylin-eosin stain; original magnification: ×400.)

The mean sebaceous gland area of psoriatic alopecia were 50% smaller than those of AA. Psoriatic alopecia had a mean surface area of 0.12 mm^2^ (*P* = .013), whereas the largest sebaceous gland in AA was 0.18 mm^2^ (*P* = .028 and *P* = .013, respectively; 1-tailed *t* test).

This study shows that there is a distinct difference in the proportion of mature to immature sebocytes between psoriatic alopecia and AA with almost 3 times more mature sebocytes in AA. It also confirms previous observations about sebaceous gland size in psoriatic alopecia and AA.^5^ Sebaceous gland in AA are 50% larger than in psoriatic alopecia. Taken together, gland size and sebocytes maturity provide a reliable histopathologic aid which is useful in distinguishing the dermal changes in AA from psoriatic alopecia. This can help prevent an overdiagnosis of AA in patients with psoriasis only. Further study of the sebaceous gland features of a larger set of cases of AA, including variants of AA, and other AA histologic simulants is warranted.^1^

## Conflicts of interest

Dr Tosti is a consultant for DS Laboratories, Monat Global, Almirall, Thirty Madison, Eli Lilly, Bristol Myers Squibb, P&G, Pfizer, Myovant. Author Nguyen and Dr Thompson have no conflicts of interest to declare.
